# Supplementary material for: The Systems Biology Research Tool: evolvable open-source software
Source: BMC Syst Biol. 2008 Jun 29;2:55. doi: 10.1186/1752-0509-2-55 (PMC2446383; doi:10.1186/1752-0509-2-55)
Supplement: Additional file 1 — SBRT Archive. An archive of the current version of the Systems Biology Research Tool. [file 1752-0509-2-55-S1.zip › sbrt-1.4.0/doc/developers_guide/api/sbrt/kernel/fba/SourceRxn.html]

SourceRxn


|  |  |  |
| --- | --- | --- |
| **Overview** \| **Package** \| **Class** \| **Use** \| **Tree** \| **Deprecated** \| **Index** \| **Help** | |  |
| **PREV CLASS**   NEXT CLASS | **FRAMES**    **NO FRAMES**     **All Classes** |  |
| SUMMARY: NESTED \| FIELD \| CONSTR \| METHOD | DETAIL: FIELD \| CONSTR \| METHOD |  |


---


## sbrt.kernel.fba Class SourceRxn

```
java.lang.Object
  sbrt.kernel.fba.ExchangeRxn
      sbrt.kernel.fba.SourceRxn
```

**All Implemented Interfaces:**: ChemRxn

---

``` public final class SourceRxn extends ExchangeRxn ```

This class is used to represent chemical species sources in Flux Balance
Analysis. A source reaction is a type of exchange reaction that supplies a
particular chemical species, but does not consume it.

Instances of this class are immutable.

**Author:**
:   This class was written and documented by Jeremiah Wright while in the
    Wagner lab.

---

**Constructor Summary**

`SourceRxn(java.lang.String chemSpecies)`
  
          Constructs a new source for the provided chemical species.


| **Method Summary** | |
| --- | --- |
| `LinearComb<java.lang.String>` | `getProducts()`             Returns a single-term linear combination. |
| `boolean` | `isEquivalent(ChemRxn rxn)`             Indicates if the provided chemical reaction is stiochiometrically equivalent to this source reaction. |
| `boolean` | `isReverse(ChemRxn rxn)`             Indicates if the provided chemical reaction is the reverse of this source reaction. |
| `java.lang.String` | `toString()`             Returns a string representation of this source reaction. |

**Methods inherited from class sbrt.kernel.fba.ExchangeRxn**

`equals, getReactants, getSpecies, hashCode`

**Methods inherited from class java.lang.Object**

`clone, finalize, getClass, notify, notifyAll, wait, wait, wait`

**Constructor Detail**

### SourceRxn

```
public SourceRxn(java.lang.String chemSpecies)
```

:   Constructs a new source for the provided chemical species.

    **Parameters:**: `chemSpecies` - the chemical species this source supplies.


**Method Detail**

### getProducts

```
public LinearComb<java.lang.String> getProducts()
```

:   Returns a single-term linear combination. The variable of this term is
    the supplied chemical species and the coefficient is 1.0.

    :   **Specified by:**: `getProducts` in interface `ChemRxn` **Overrides:**: `getProducts` in class `ExchangeRxn`
    :   **Returns:**: a single-term linear combination. The variable of this term is the supplied chemical species and the coefficient is 1.0.

---


### isEquivalent

```
public boolean isEquivalent(ChemRxn rxn)
```

:   Indicates if the provided chemical reaction is stiochiometrically
    equivalent to this source reaction. Source reactions can only be
    equivalent to other sources (of type `SourceRxn`). Two chemical
    species sources are equivalent only if they supply the same chemical
    species.

    :   **Parameters:**: `rxn` - the chemical reaction to test for equivalence. **Returns:**: `true` if both reactions are equivalent, `false` otherwise.

---


### isReverse

```
public boolean isReverse(ChemRxn rxn)
```

:   Indicates if the provided chemical reaction is the reverse of this source
    reaction. The only possible reverse reactions of source reactions are
    sink reactions (SinkRxn). A source and sink are the
    reverse of each other only if their associated chemical species is the
    same.

    :   **Parameters:**: `rxn` - the chemical reaction to test. **Returns:**: `true` if the provided reaction is the reverse of this reaction, `false` otherwise.

---


### toString

```
public java.lang.String toString()
```

:   Returns a string representation of this source reaction.

    :   **Specified by:**: `toString` in interface `ChemRxn` **Overrides:**: `toString` in class `java.lang.Object`
    :   **Returns:**: a string representation of this source reaction.


---


|  |  |  |
| --- | --- | --- |
| **Overview** \| **Package** \| **Class** \| **Use** \| **Tree** \| **Deprecated** \| **Index** \| **Help** | |  |
| **PREV CLASS**   NEXT CLASS | **FRAMES**    **NO FRAMES**     **All Classes** |  |
| SUMMARY: NESTED \| FIELD \| CONSTR \| METHOD | DETAIL: FIELD \| CONSTR \| METHOD |  |


---
